# Environmental Regulation of PndbA600, an Auto-Inducible Promoter for Two-Stage Industrial Biotechnology in Cyanobacteria

**DOI:** 10.3389/fbioe.2020.619055

**Published:** 2021-01-19

**Authors:** Mary Ann Madsen, Graham Hamilton, Pawel Herzyk, Anna Amtmann

**Affiliations:** ^1^College of Medical, Veterinary and Life Sciences, Institute of Molecular, Cell and Systems Biology, University of Glasgow, Glasgow, United Kingdom; ^2^Glasgow Polyomics, Wolfson Wohl Cancer Research Centre, University of Glasgow, Glasgow, United Kingdom

**Keywords:** cyanobacteria, biotechnology, two-stage cultivation strategy, stationary phase, promoter, transcriptomics, nutrient limitation

## Abstract

Cyanobacteria are photosynthetic prokaryotes being developed as sustainable platforms that use renewable resources (light, water, and air) for diverse applications in energy, food, environment, and medicine. Despite the attractive promise that cyanobacteria offer to industrial biotechnology, slow growth rates pose a major challenge in processes which typically require large amounts of biomass and are often toxic to the cells. Two-stage cultivation strategies are an attractive solution to prevent any undesired growth inhibition by de-coupling biomass accumulation (stage I) and the industrial process (stage II). In cyanobacteria, two-stage strategies involve costly transfer methods between stages I and II, and little work has been focussed on using the distinct growth and stationary phases of batch cultures to autoregulate stage transition. In the present study, we identified and characterised a growth phase-specific promoter, which can serve as an auto-inducible switch to regulate two-stage bioprocesses in cyanobacteria. First, growth phase-specific genes were identified from a new RNAseq dataset comparing two growth phases and six nutrient conditions in *Synechocystis* sp. PCC 6803, including two new transcriptomes for low Mg and low K. A type II NADH dehydrogenase (*ndbA*) showed robust induction when the cultures transitioned from exponential to stationary phase growth. Behaviour of a 600-bp promoter sequence (PndbA600) was then characterised in detail following the expression of PndbA600:GFP in *Synechococcus* sp. PCC 7002. Culture density and growth media analyses showed that PndbA600 activation was not dependent on increases in culture density *per se* but on N availability and on another activating factor present in the spent media of stationary phase cultures (Factor X). PndbA600 deactivation was dependent on the changes in culture density and in either N availability or Factor X. Electron transport inhibition studies revealed a photosynthesis-specific enhancement of active PndbA600 levels. Our findings are summarised in a model describing the environmental regulation of PndbA600, which can now inform the rational design of two-stage industrial processes in cyanobacteria.

## Introduction

Cyanobacteria are being developed as sustainable platforms that use renewable resources (light, water, and air) for diverse industrial applications, including the manufacturing of commodity and high-value products and remediation of heavy metals or salt (Amezaga et al., 2014; Al-Haj et al., 2016; Singh et al., 2016, 2017; Miao et al., 2020). This phylum of oxygenic photosynthetic bacteria inhabits virtually every niche across the planet and, coupled with its metabolic plasticity, lends itself to a vast variety of industrial settings and processes (Thajuddin and Subramanian, 2005; Xiong et al., 2017). Cyanobacteria produce a diverse palette of natural products with applications in energy, food, environment, and medicine, and are easily engineered for recombinant production (Camsund and Lindblad, 2014; Dittmann et al., 2015; Singh et al., 2017).

Despite the attractive promise that cyanobacteria offer to industrial biotechnology, they present unique challenges which have hampered its adoption by an industry currently dominated by well-established heterotrophic systems, such as *Escherichia coli* and *Saccharomyces cerevisiae*. While similarly amenable to high-throughput screening and engineering, many cyanobacterial strains still pose key technical difficulties such as cultivation and transformation—issues that have long been optimised for their heterotrophic competitors. With the collection of data and progress in technology, these barriers are gradually coming down. For instance, energetic and economic costs of cultivation and product purification are alleviated by rapid developments in photobioreactor and downstream processing technologies (Pierobon et al., 2018). Substantial effort has also been directed towards the development of molecular tools to genetically engineer these photosynthetic prokaryotes in which both standard prokaryote and photosynthetic eukaryote toolboxes are ineffective (Berla et al., 2013; Camsund and Lindblad, 2014; Santos-Merino et al., 2019). Despite the improvements in cultivation systems and the identification of relatively fast-growing cyanobacterial strains, however, slow growth rates continue to pose a major challenge to cyanobacterial biotechnology (Gale et al., 2019).

Industrial applications typically require a large biomass to obtain sufficient productivity. It is, therefore, important to avoid any growth inhibition during biomass accumulation in order to generate the optimal biomass as quickly as possible. In addition, computational analyses indicate trade-offs between biomass production and product synthesis in cyanobacteria (Knoop and Steuer, 2015). Two-stage cultivation strategies are, therefore, an attractive approach to decouple the industrial process (stage II) from biomass accumulation (stage I). Growth inhibition is thus minimised by alleviating issues arising from product/process toxicity, and stage I growth rates/stage II productivity is maximised by preferentially allocating resources (carbon precursors, ATP energy, and NAD(P)H reducing power) to growth or productivity, respectively (Burg et al., 2016).

In cyanobacteria, two-stage cultivation strategies typically require extra steps between stages I and II that add monetary and energetic costs to the process. The most common approach involves physical transfer of cultures from stage I to stage II, promoting conditions using centrifugation, filtration, flocculation, or sedimentation (Monshupanee et al., 2016; Kushwaha et al., 2018; Testa et al., 2019; Aziz et al., 2020). Alternatively, stage II can be induced by the application of physical and/or chemical stimuli such as changing light conditions for pigment production or temperature and salt stress for polysaccharide production in *Spirulina* (Lee et al., 2012, 2016). Strategies which eliminate these extra steps between stages I and II can greatly improve the economic feasibility of these systems.

Batch grown systems are well-suited for two-stage approaches. Bacterial growth is characterised by three successive phases: lag phase, exponential growth phase, and stationary phase. Cyanobacterial batch cultures show a similar growth pattern with the exception of an extended growth phase comprised of a shorter early growth phase, from which exponential growth rates are commonly reported, and a longer late growth phase, often termed the linear growth phase, as the cultures transition to the stationary phase (Schuurmans et al., 2017). Inherent differences between the growth and stationary phases of batch systems can be used to regulate two-stage processes and initiate stage II once maximum culture density has been achieved in the late growth/early stationary phase neither with any manipulation of culture nor with any added cost to the process.

Promoters are regulatory elements in the DNA that function as biological switches. Ideally, promoters controlling two-stage processes should be inactive during stage I and become active at the onset of stage II (Supplementary Figure S1). Auto-inducible promoters, which respond to endogenous signals, have the distinct advantage of not requiring any additional supplements, thus simplifying and improving the sustainability of the process. In the case of two-stage processes, promoters that specifically respond to changes in growth phase, particularly the transition to stationary phase, are ideal candidates. Libraries of stationary phase promoters have been developed for *E. coli* (Miksch et al., 2005). While orthogonal promoters derived from other organisms are generally preferred in order to avoid interference of engineered systems by host machinery, well-established prokaryotic tools perform poorly in cyanobacteria (Huang et al., 2010). In cyanobacteria, several growth phase-responsive genes and promoters have been reported for some model strains (Ludwig and Bryant, 2011; Berla and Pakrasi, 2012; Kopf et al., 2014; Ruffing et al., 2016). However, we still lack a detailed understanding of activation/deactivation behaviours and of performance across strains.

**FIGURE 1:**
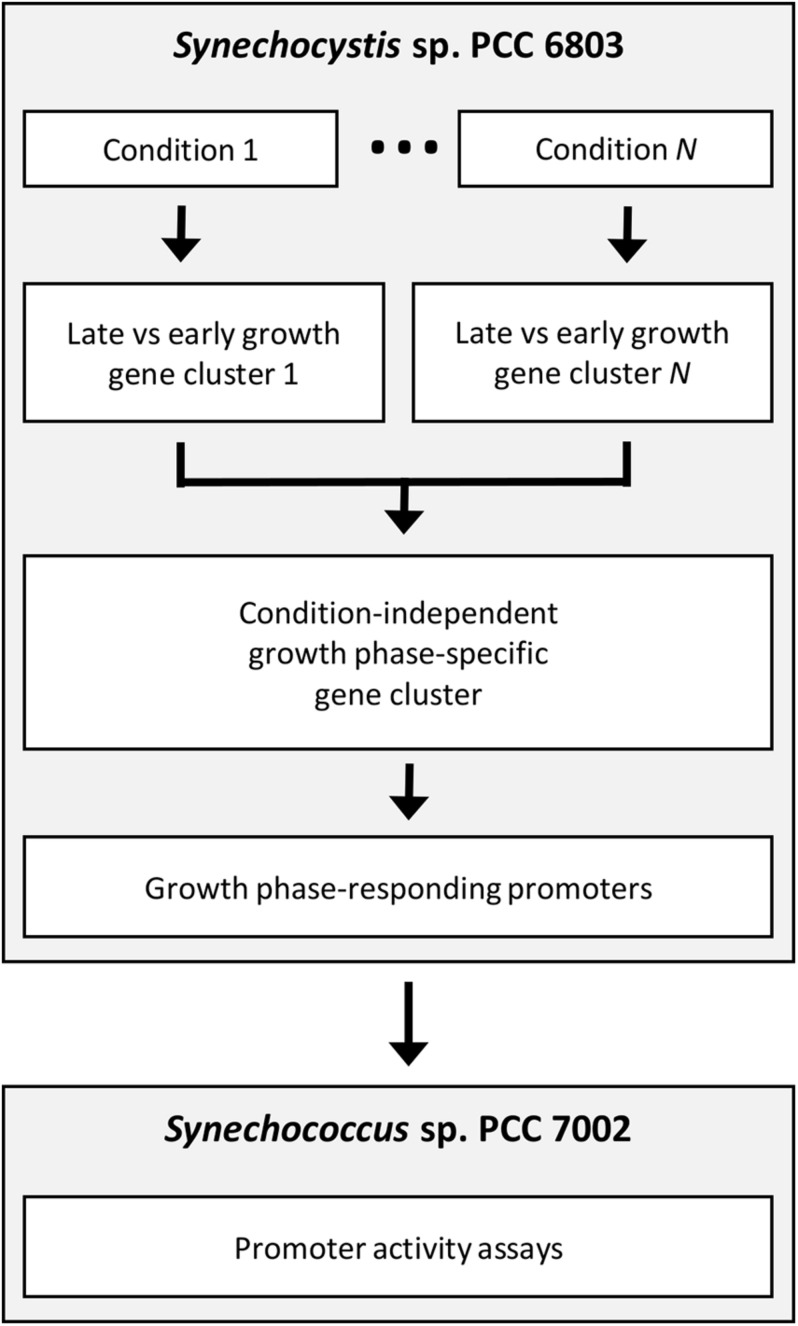
Workflow for the identification and characterisation of growth phase-specific promoters. *Synechocystis* sp. PCC 6803 was cultivated in numerous different conditions. RNA sequencing of samples harvested during early and late growth phases led to the identification of condition-independent, growth phase-specific genes and candidate promoters. Heterologous promoter behaviour was characterised using a green fluorescent protein (GFP)-based assay in *Synechococcus* sp. PCC 7002.

In this study, we aimed to identify and characterise an auto-inducible promoter for two-stage batch cultivation strategies in cyanobacteria using the approach presented in Figure 1. Two different species of cyanobacteria were used to avoid potential issues with genetic instability or cross-talk between native expression machinery and engineered expression systems (Camsund and Lindblad, 2014; Gordon and Pfleger, 2018). First, RNA sequencing analyses comparing transcriptional profiles across growth phases and nutrient conditions led to the identification of robust growth phase-specific genes and thus candidate promoters in the freshwater cyanobacterium *Synechocystis* sp. PCC 6803. Next, the behaviour of a promoter, PndbA600, was characterised in response to changing culture density, growth media, and cellular redox status using a green fluorescent protein (GFP) assay in *Synechococcus* sp. PCC 7002. Our findings are summarised in a model describing the environmental regulation of PndbA600, which can inform the rational design of sustainable, two-stage industrial processes in cyanobacteria.

## Materials and Methods

### Strains and Culture Conditions

#### Cyanobacterial Strains

*Synechocystis* sp. PCC 6803 was grown photoautotrophically in Dreschel flasks in water baths equilibrated to 30°C with photoperiod 12 h/12 h light/dark and light intensity 80 μmol photons m^–2^ s^–1^. *Synechococcus* sp. PCC 7002 was grown photoautotrophically in Bijou bottles maintained at 30°C in a walk-in environmental growth chamber (Conviron model MTPS72) with photoperiod 16 h/8 h light/dark and light intensity 150 μmol photons m^–2^ s^–1^. Cultures had a working volume of 60–75% relative to the culture vessel capacity, which were illuminated with fluorescent cool white lights and sparged with humidified ambient air.

#### Growth Media

For control conditions, *Synechocystis* cultures were grown in BG11 medium (Stanier et al., 1971) and *Synechococcus* cultures were grown in A + medium (Stevens and Porter, 1980). For low nutrient conditions, individual nutrients (N, P, K, S, and Mg) were reduced to the indicated concentrations relative to the concentration in control BG11 or A + medium, and counter ions were replaced with control concentrations of KCl, MgCl, Na_2_SO_4_ or NaH_2_PO_4_ (see Supplementary Tables S1 and S2 respectively). To obtain “spent” media, the supernatant of stationary phase *Synechococcus* cultures (cultivated for ≥ 5 weeks) was harvested after centrifugation at 4,000 *g* for 20 min at room temperature, and filter-sterilised.

#### Culture Setup

Twenty millilitre control medium was inoculated with strains maintained as DMSO stocks at −80°C or on solid media maintained at 23°C. For control and low nutrient conditions, 20 ml cultures were grown to optical density (OD) 1–5, diluted to OD 1 in the relevant growth medium and 0.5 ml was used to inoculate 150 ml in the relevant growth medium. For promoter activation and deactivation experiments, 750 and 150 ml cultures were grown in control conditions to low density (OD < 5, GFP < 300) and high density (OD ≥ 12, GFP > 850), respectively. Cells were harvested by centrifugation at 4,000 *g* for 20 min at room temperature, the supernatant was removed, and the pellets were washed and resuspended at OD 1 or 12 (for low and high density, respectively) in the relevant growth medium.

#### Electron Transport Inhibitors

3-(3,4-dichlorophenyl)-1,1-dimethylurea (DCMU, aka Diuron; Sigma) or malonic acid (Sigma) were added at the indicated concentrations to either young (1-week old cultures, OD < 5, GFP < 300) or mature (4-week old cultures, OD ≥ 12, GFP > 850) 150 ml cultures grown in control conditions.

#### Growth Monitoring

Growth was monitored by measuring OD at 730 nm (OD_730_) within the linear range (OD 0.05–1.00) of a Lambda 45 UV/VIS Spectrophotometer (PerkinElmer).

### RNA Analyses

Total RNA was extracted using the RNeasy Mini Kit (Qiagen, Venlo, Netherlands). Frozen cell pellets were resuspended in 700 μl Buffer RLT and cells were disrupted using 0.5 g of 0.5 mm diameter glass beads for 5 min at 30 Hz in a TissueLyser (Qiagen, Venlo, Netherlands). Following centrifugation at 10,000 *g* for 1 min, the supernatant was applied to the RNeasy spin column and RNA purified as recommended by the supplier.

For RNA sequencing, messenger RNA (mRNA) was enriched using the MICROBExpress Kit (Ambion, Austin, TX, United States). RNA quality was assessed before and after mRNA enrichment using an Agilent^®^ 2100 Bioanalyzer^TM^. Complementary DNA (cDNA) libraries were generated using TruSeq Stranded mRNA Library Prep Kit (Illumina) and sequenced using the Illumina MiSeq System at Glasgow Polyomics. Reads were processed and mapped to the *Synechocystis* genome (GenBank assembly accession GCA_000009725.1) using TopHat and Cuffdiff software (Trapnell et al., 2012). A total of 640,131,273 reads were obtained with average reads of 16.4 million reads per sample and an average length of 75 base pairs. A total of 546,270,878 reads (85.3%) were mapped to the genome with a tolerance of a 2 base pair mismatch. The number of reads mapped to each coding sequence was calculated and normalised for gene length (number of fragments mapped per kilobase of gene) and library depth (total number of aligned reads in the experiment). Data are thus presented as fragments per kilobase of gene per million reads mapped (FPKM).

For each condition and time point, replicate samples from three independently grown cultures were sequenced, resulting in a total of 39 RNAseq datasets (1 control condition × 3 time points × 3 replicates + 5 low nutrient conditions × 2 time points × 3 replicates). The raw RNA sequencing data are available from the European Nucleotide Archive, accession number PRJEB40560. Significant differences between conditions and time points were determined using Cuffdiff software (Trapnell et al., 2012). Multi-dimensional scaling of the RNA sequencing data was performed using the CummeRbund visualisation package (Trapnell et al., 2012).

For quantitative real-time PCR (qPCR), total RNA was isolated from different cultures to those used for RNA sequencing experiments, resulting in a total of 36 qPCR samples (6 nutrient conditions × 2 time points × 3 replicates). cDNA libraries were generated using random primers with the QuantiTect Reverse Transcription Kit (Qiagen, Venlo, Netherlands). qPCR was performed in a StepOnePlus Real-Time PCR System (Life Technologies). Primers were designed to amplify 110–155 bp products with a 60°C annealing temperature (Table 1) using Primer 3 software (Untergasser et al., 2012) and interrogated using Brilliant III Ultra-Fast SYBR Green QPCR Master Mix (Agilent). Primer pair efficiencies were determined using serial dilutions of gel purified RT-PCR product. Standard curves were included in all qPCR runs to transform threshold cycles into RNA concentrations, which were ratioed against the internal control *slr0211*. For replication, assays were performed using cDNA from three independently grown cultures. Significant differences between conditions and time points were determined by two-way ANOVA with Tukey (HSD) *post-hoc* analysis using SigmaPlot software (Systat). Negative (no template) controls were included and a melting curve analysis was performed in all assays.

**TABLE 1:** Primer sequences for quantitative real-time PCR.

Target	Forward primer sequence	Reverse primer sequence
gene	(5′–3′)	(5′–3′)
*slr0211*	CCTGCTCCGGGCCTTGG	CTGGTATTGAATGGGGCCAC
*slr0451*	GAACAACAGGCCAGGGTAG	CGTAGTTCTTGCCGTTGGTG
*sll0401*	GAGAGTAGAAGCCGTTACCC	GCTGACGGAGAAGGAGCC
*slr1697*	CCCGATTTAACACCAATGTCC	GACTCAATATTGCTGGTAGCC
*slr0073*	GGAATATTGCACTCGTCTGGG	GCCAAGGTACGGTAGGAATG
*slr2144*	CAACAGTGACGGTCTGACC	CACCACTGCTTGCCCATCC
*psbA1*	CCTGTGGTCACGGTTCTGTT	TGCCATCAATATCCACCGGG
*rnpB*	GTGAGGACAGTGCCACAGAA	GATACTGCTGGTGCGCTCTT
*ndbA*	GACAAAAACGGTGCTCTGGG	CTCAAATCCGGGTTGACCAC
*slr1747*	GTTGCCCTCCCCTTGGTG	GAATATGGCTCGAATCCAACAC

### Promoter Analyses

A 600 bp sequence occurring directly upstream of the *ndbA* gene, PndbA600, was amplified from *Synechocystis* genomic DNA using primers containing BioBrick prefix and suffix overhangs for cloning into the pAQ1BB transformation vector: forward primer 5′- gaattcgcggccgcttctagagTTAATGGATCGTTACCATTCCCAC -3′ and reverse primer 5′- ctgcagcggccgctactagtaAGCAACGGCG AAAATATTACGATTTG-3′. Following sequence confirmation in the pGEM-T Easy vector, PndbA600 was cloned upstream of a synthetic RBS-reporter construct comprised of RBS3 and GFP BioBrick part BBa_E0040 in the pAQ1BB vector (Madsen et al., 2018) to generate pAQ1BB:PndbA600:RBS3:GFP (Supplementary Figure S2). The sequence of the promoter-reporter construct is presented in Supplementary Figure S3. The promoter-GFP construct was integrated into a neutral site in the *Synechococcus* genome by natural transformation and verified by PCR amplification and sequencing to generate the transgenic *Synechococcus* PndbA600:GFP strain. GFP was measured at regular intervals during culture growth by adjusting culture density to OD_730_ 0.25–0.30 and measuring fluorescence using 480 nm excitation and 514 nm emission wavelengths using a LS 55 Luminescence Spectrophotometer (PerkinElmer, Waltham, MA, United States).

## Results

### Environmental Conditions to Control Growth Phase Transition in *Synechocystis* Batch Cultures

**FIGURE 2:**
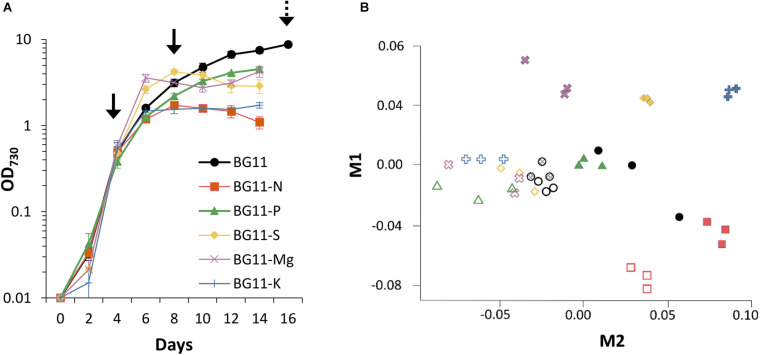
Growth and transcriptomics of *Synechocystis* sp. PCC 6803. **(A)** Culture density (OD_730_) of *Synechocystis* batch cultures cultivated under control (BG11) and low nutrient conditions (12.5% N, P, S, Mg, or K in BG11 background, Supplementary Table S1). Solid arrows show when samples were collected for RNA sequencing from all conditions. Dashed arrow indicates additional samples collected from control conditions. Data are means ± S.E.M. of three independent cultures. **(B)** Multi-dimensional scaling plot of 39 RNAseq expression profiles. Distances between points reflect relative similarities between samples based on genome-wide transcript levels. Symbols and colours are same as in **(A)**. Open symbols represent early growth phase samples (day 4). Closed symbols represent late growth phase samples (day 8 in low nutrient and day 16 in control conditions). Hatched circles represent control samples harvested on day 8.

Stationary phase can be induced by many different factors including nutrient limitation, toxic by-product accumulation, or a variety of stress factors such as pH or temperature (Nyström, 2004). For robust separation of general responses to changes in the growth phase from specific responses to individual factors, several different conditions inducing transition to stationary phase were required. Nutrient deficiencies limit the growth of cyanobacteria (Hirani et al., 2001; Richaud et al., 2001) and were used here to induce the stationary phase. *Synechocystis* sp. PCC 6803 was cultivated under control conditions (BG11) and low levels of individual nutrients as reported before, with 12.5% N, P, or S in BG11 background. In addition, two new low nutrient conditions were tested, 12.5% Mg and 12.5% K in BG11 background. Nutrients are co-supplied with a counter ion as an electrically neutral salt, so counter ions were replaced up to the control concentration (Supplementary Table S1). Figure 2A shows that *Synechocystis* sp. PCC 6803 batch cultures cultivated in all five low nutrient conditions transition to the stationary phase at an earlier time (day 8) and lower density (OD_730_ 1.5–4.2) compared to the control condition (day 16, OD_730_ 8.7). Thus, low nutrient conditions were used to induce early transition to the stationary phase in response to specific environmental stimuli.

### RNA Sequencing of Early and Late Growth Phases in *Synechocystis*

To compare the transcriptomes of early and late growth phases, time points were selected for RNA sequencing based on the growth curves (black arrows in Figure 2A). Early samples were harvested during the exponential growth phase (day 4), and late samples were harvested as the cultures transitioned into the stationary phase (day 8). To account for later transition to the stationary phase, control cultures were also harvested on day 16. For replication, three cultures were independently grown in each condition. RNA purification and sequencing is described in Materials and Methods (Section “RNA Analyses”), and the normalised transcript levels for all genes in all 39 samples, together with statistical parameters, are available in Supplementary Table S3. Transcript counts are presented as FPKM.

Figure 2B shows a multi-dimensional scaling plot based on normalised FPKM values in the 39 RNAseq samples. Early samples generally group together with the exception of BG11-N, suggesting a distinct early response to low N. Additionally, samples harvested on day 8 under control conditions cluster with early samples, reflecting that nutrients were not yet limiting in this condition and control cultures were still in the early growth phase. Late samples separate according to condition with close grouping of replicates, demonstrating nutrient-specific transcriptional responses. Greater variation is observed in late control samples, possibly due to simultaneous limitation of multiple nutrients in the optimised BG11 medium in which nutrients deplete at equal rates.

### Robust, Late Growth Phase-Specific Genes of *Synechocystis*

To identify condition-independent, growth phase-responsive genes, we looked for genes that were upregulated in the late growth phase compared to the early growth phase in all of the conditions tested. Genes were, therefore, selected from the RNA sequencing dataset based on a significance value of *p* < 0.05 between early and late samples within each condition and log_2_(late/early) > 1 for all six conditions. Table 2 presents 24 late growth phase-specific genes from the *Synechocystis* RNA sequencing dataset. The majority of genes are annotated as hypothetical proteins (16/24, 67%); however, there are also genes with annotated functions in cell killing, energy metabolism, photosynthesis and respiration, regulatory functions, and transport.

**TABLE 2:** Late growth phase-specific genes of *Synechocystis* sp. PCC 6803.

Gene ID	Gene symbol	Gene product	Functional category
*slr1747*		Cell death suppressor protein Lls1 homologue	Cellular processes
*slr2132*		Phosphotransacetylase	Energy metabolism
*sll0549*		Hypothetical protein	Hypothetical
*sll0528*		Hypothetical protein	Hypothetical
*slr1119*		Hypothetical protein	Hypothetical
*sll1675*		Hypothetical protein	Hypothetical
*sll1355*		Hypothetical protein	Hypothetical
*sll1274*		Hypothetical protein	Hypothetical
*sll1769*		Hypothetical protein	Hypothetical
*sll1158*		Hypothetical protein	Hypothetical
*sll1884*		Hypothetical protein	Hypothetical
*slr1674*		Hypothetical protein	Hypothetical
*slr0959*		Hypothetical protein	Hypothetical
*slr0292*		Hypothetical protein	Hypothetical
*sll6052*		Hypothetical protein	Hypothetical
*sll6053*		Hypothetical protein	Hypothetical
*sll6054*		Hypothetical protein	Hypothetical
*sll6055*		Hypothetical protein	Hypothetical
*slr1498*	*hypD*	Putative hydrogenase protein HypD	Other categories
*slr1675*	*hypA1*	Putative hydrogenase protein HypA1	Other categories
*slr0851*	*ndbA*	Type 2 NADH dehydrogenase	Photosynthesis and respiration
*slr0741*		Transcriptional regulator	Regulatory functions
*slr0096*		Low affinity sulphate transporter	Transport and binding proteins
*slr0529*	*ggtB*	Glucosylglycerol transport system substrate-binding protein	Transport and binding proteins

**FIGURE 3:**
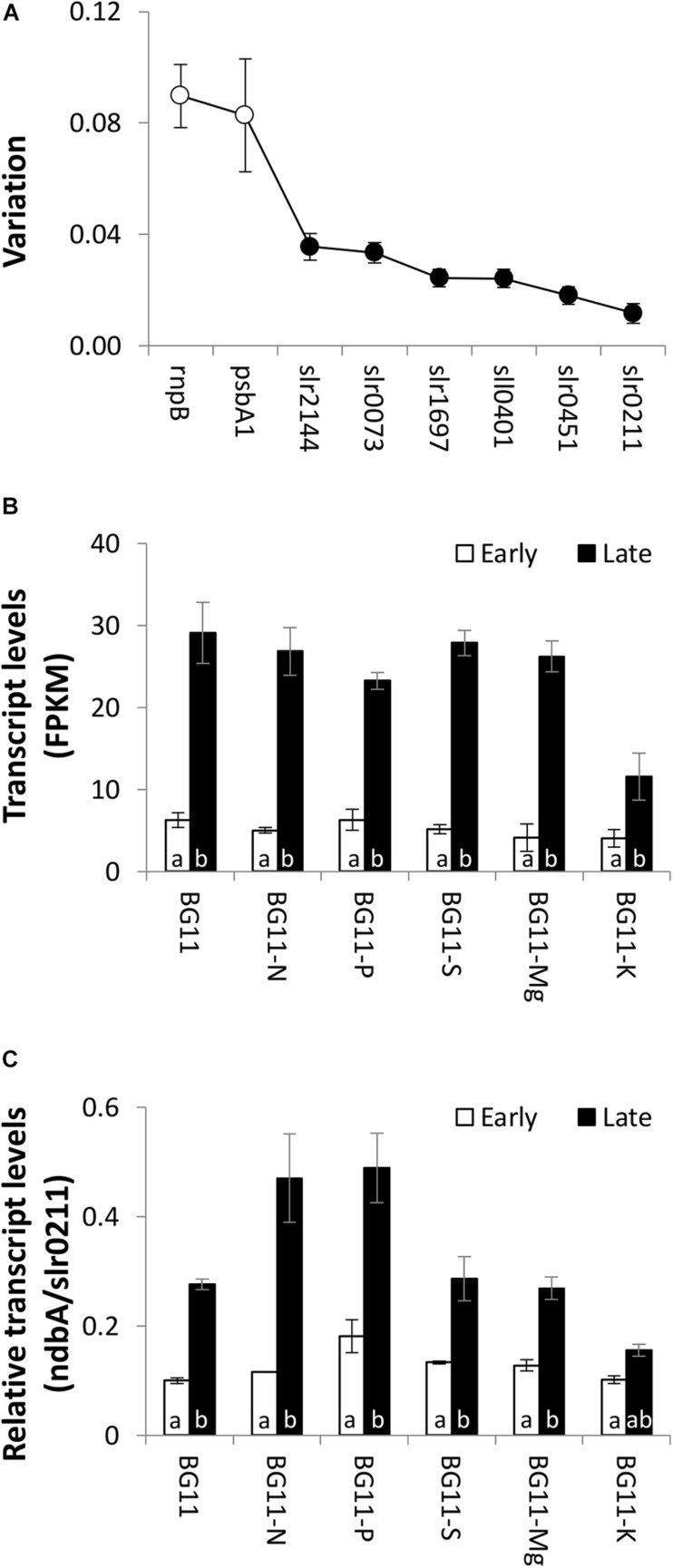
Native *ndbA* transcript levels. **(A)** Variation of Ct values determined using NormFinder software (Andersen et al., 2004). qPCR was performed with 36 samples representing the early and late growth phases of three independent cultures of *Synechocystis* cultivated under control (BG11) and low nutrient conditions (12.5% N, P, S, Mg, or K in BG11 background). White circles represent reference genes used in other studies (Foster et al., 2007; Pinto et al., 2012). Black circles represent genes selected from the RNA sequencing dataset based on low standard error. Data are means ± S.E.M. of three independent cultures. *slr0211* was selected as reference gene for further experiments. **(B,C)** Transcript levels of *ndbA* determined by **(B)** RNA sequencing (normalised to gene length and read counts as FPKM) and **(C)** qPCR (normalised to *slr0211*) in the early (white bars) and late (black bars) growth phases of *Synechocystis* cultivated under control (BG11) and low nutrient conditions (12.5% N, P, S, Mg, or K in BG11 background). Data are means ± S.E.M. of three independent cultures. Different letters indicate significant difference across all conditions (*p* < 0.05; two-way ANOVA using Tukey (HSD) *post-hoc* analysis).

RNAseq expression profiles were verified using real-time qPCR. Three new *Synechocystis* cultures were grown for each of the six nutrient conditions, and samples were harvested during the early and late growth phases (36 samples total). To control for potential biases introduced during mRNA enrichment for the RNA sequencing analyses, cDNA for qPCR analyses was generated from total RNA. *slr0211* (encoding a hypothetical protein) was selected as a reference gene for qPCR normalisation based on low standard error of FPKM across the 39 RNAseq samples and low variation of Ct values across the 36 qPCR samples (Figure 3A). While there were small differences in the level of upregulation within individual conditions, there was an overall excellent agreement between RNAseq and qPCR expression profiles for *ndbA* (*slr0851*, encoding a type II NADH dehydrogenase, Figures 3B,C) and *slr1747* (encoding a homologue of the cell death suppressor protein Lls1, Supplementary Figure S4). While these genes are adjacent to one another on the *Synechocystis* chromosome, they are independent transcriptional units (Kopf et al., 2014), and both showed significantly higher transcript levels in the late growth phase compared to the early growth phase in all six nutrient conditions. The level of *ndbA* upregulation was relatively consistent across the conditions tested; therefore, this gene was selected for promoter analyses.

### Heterologous Activity of the *ndbA* Promoter, PndbA600, in *Synechococcus*

**FIGURE 4:**
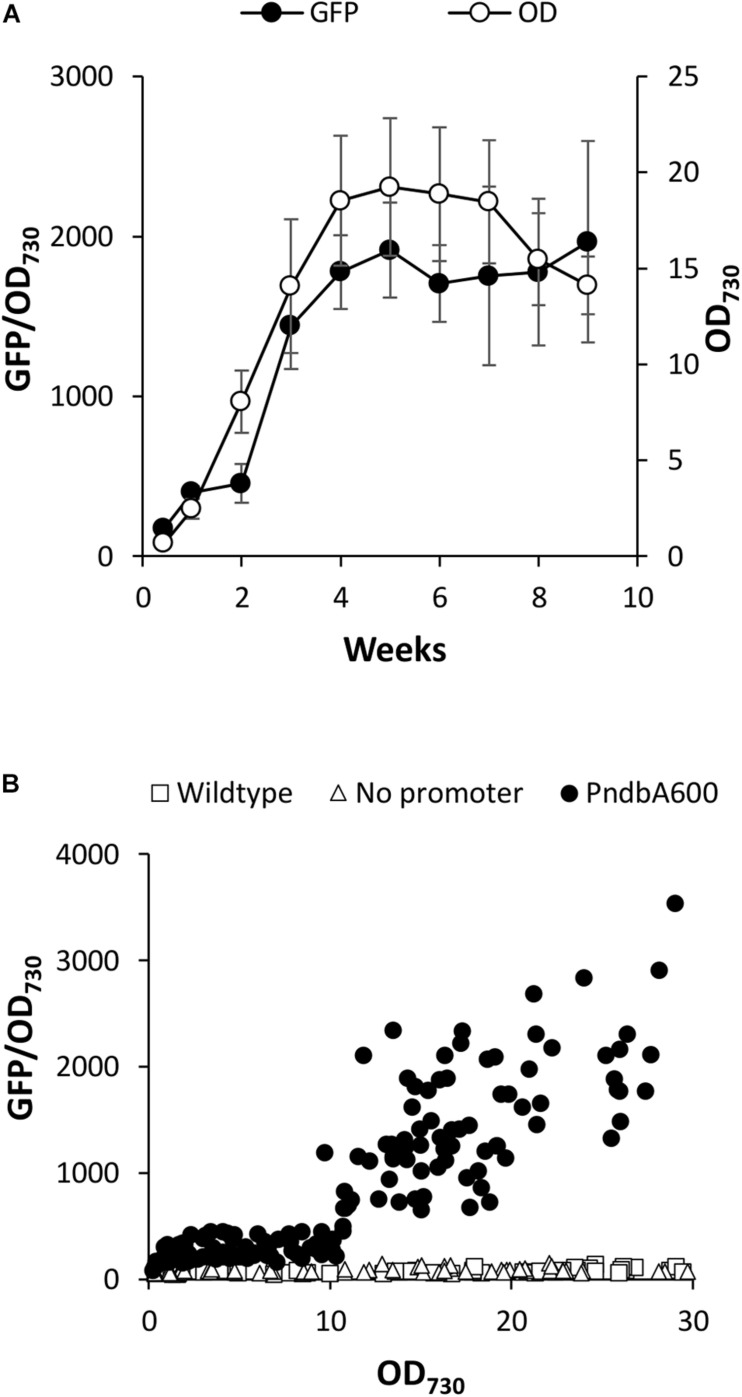
Heterologous PndbA600 activity under control conditions. **(A)** Promoter activity (black circles, GFP fluorescence normalised to OD_730_, left axis) in *Synechococcus* PndbA600:GFP. Culture density (OD_730_, right axis) is shown with white circles. Data are means ± S.E.M. of three independent cultures grown in control A + media. **(B)** Promoter activity (GFP fluorescence normalised to OD_730_) against culture density (OD_730_) of *Synechococcus* cultures grown in control conditions. Data are shown for the following lines: wild-type (no GFP, squares, *n* = 7), no promoter (*Synechococcus* transformed with GFP, triangles, *n* = 6), *Synechococcus* expressing GFP under the control of PndbA600 (*Synechococcus* PndbA600:GFP, circles). Measurements taken after maximum culture density was achieved are not shown.

Heterologous systems have several advantages for the characterisation of molecular tools for metabolic engineering. For example, the interference from native regulatory machinery is minimised, such as small RNAs important for adaptation to changing environments in cyanobacteria (Hu and Wang, 2018). Furthermore, introduction of high copy numbers of an endogenous, or even homologous, promoter could outcompete the native promoter (or vice versa) and potentially cause genetic instability in cyanobacteria (Gordon and Pfleger, 2018). Although there is a potential for common regulatory machinery, such as transcription factors across cyanobacteria, *Synechocystis* promoters are routinely used to drive the heterologous expression in *Synechococcus* sp. (Huang et al., 2010; Wang et al., 2012). We therefore used an established GFP-based promoter assay in *Synechococcus* sp. PCC 7002 (Madsen et al., 2018), to assess whether the *ndbA* promoter controls growth phase-specific transcription. For this, we analysed the 600 bp sequence directly upstream of the *ndbA* start codon, which, in addition to the core promoter, includes the 5′UTR and other potential genetic features. This sequence was designated PndbA600 and cloned upstream of a GFP reporter gene and integrated into a neutral site in the *Synechococcus* genome. Following confirmation by PCR and sequencing, the transgenic strain *Synechococcus* PndbA600:GFP was grown in A + (control) medium, and GFP fluorescence per cell (GFP normalised to OD_730_ of the GFP sample) was measured throughout culture growth. Figure 4A shows that under control conditions the OD_730_-normalised GFP fluorescence was low during the early stages of growth followed by a sharp increase in week 3, concomitant with transition to the late growth phase. High levels were then maintained over several weeks of the stationary phase. Notably, PndbA600 showed two distinct levels of the promoter activity with low activity at low density (OD < 10.7, GFP < 450) and high activity at high density (OD > 10.75, GFP 636-3590; Figure 4B). The GFP signal reflects PndbA600 promoter activity: background fluorescence levels in the wild-type and no promoter controls were lower than those observed in *Synechococcus* PndbA600:GFP (GFP < 120). These combined results show that the 600 bp upstream sequence of *ndbA*, PndbA600, shows both growth phase- and culture density-specific activity in *Synechococcus* sp. PCC 7002 grown in control conditions.

### Effect of Culture Density on PndbA600 Activity

Culture density, possibly due to direct cell-to-cell interactions, has been suggested as a factor controlling cell division and the transition into stationary phase in cyanobacteria (Esteves-Ferreira et al., 2017). Additional factors could include self-shading and thus light limitation in higher density cultures or extracellular metabolites e.g., signalling molecules secreted to the growth medium (Abisado et al., 2018; Clark et al., 2018). To investigate the relationship between culture density and promoter activity in more detail, we attempted to separate the effect of culture density from the effect of the growth medium. For this experiment, *Synechococcus* PndbA600:GFP cultures were grown to either low density to investigate PndbA600 activation or high density to investigate PndbA600 deactivation. Culture density was then modulated by harvesting the cells and resuspending them at either low or high density. Growth media were modulated by resuspending the cells in either a fresh (control) medium or a spent medium harvested from stationary phase cultures.

**FIGURE 5:**
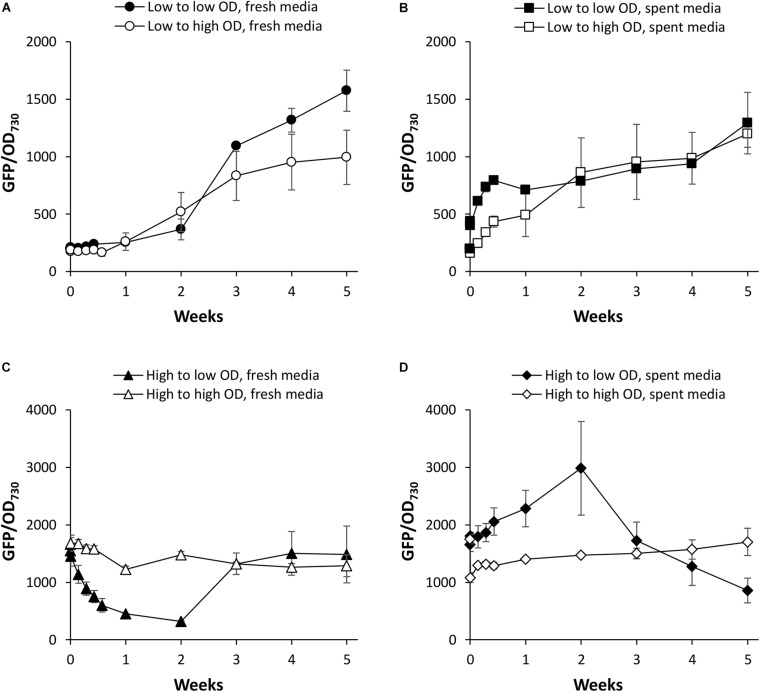
The effect of culture density on PndbA600 activity. PndbA600 activity (GFP fluorescence normalised to OD_730_) in *Synechococcus* PndbA600:GFP cultures pre-grown to certain density and resuspended to another density at time point 0. Cultures shown in **(A,B)** were started from young, low density cultures with low promoter activity (OD < 5, GFP < 300). Cultures shown in **(C,D)** were started from mature, high density cultures with high promoter activity (OD > 12, GFP > 850). Cultures were resuspended to low (black symbols) or high (white symbols) density in fresh control media **(A,C)** or spent media of stationary phase cultures **(B,D)**. Data are means ± S.E.M. of three independent cultures. Accompanying growth curves are presented in Supplementary Figure S5.

We first investigated PndbA600 activation using young, low density cultures with low promoter activity. Figure 5A shows that increasing culture density from low to high OD is not sufficient to activate PndbA600. GFP fluorescence only increased after a period of growth in the fresh medium for both low to low and low to high OD cultures (see also Supplementary Figure S5A). By contrast, resuspending low density cultures in the spent medium led to rapid PndbA600 activation with faster activation in cultures resuspended at low density compared to high density (Figure 5B and Supplementary Figure S5B). These results suggest that PndbA600 activation is not dependent on high culture density *per se* but requires one or more components of the spent stationary phase medium, designated as Factor X.

PndbA600 deactivation was then investigated using mature, high density cultures with high promoter activity. Figure 5C shows that decreasing culture density from high to low OD in a fresh medium resulted in a rapid loss of GFP fluorescence (within 1 week), indicating rapid deactivation of PndbA600. The promoter then regained activity as the culture moved again into the late growth phase (see also Supplementary Figure S5C). PndbA600 deactivation requires the decrease in cell density: mature, high density cultures resuspended at high density in fresh media maintained high promoter activity. PndbA600 deactivation also requires the fresh growth medium: mature, high density cultures resuspended at low density in spent media maintained high promoter activity (Figure 5D and Supplementary Figure S5D). This suggests that the alleviation of Factor X from the spent stationary phase medium is a co-requirement for PndbA600 deactivation. In summary, PndbA600 deactivation requires both low cell density and fresh growth medium.

### The Effect of Nutrient Availability on PndbA600 Activity

**FIGURE 6:**
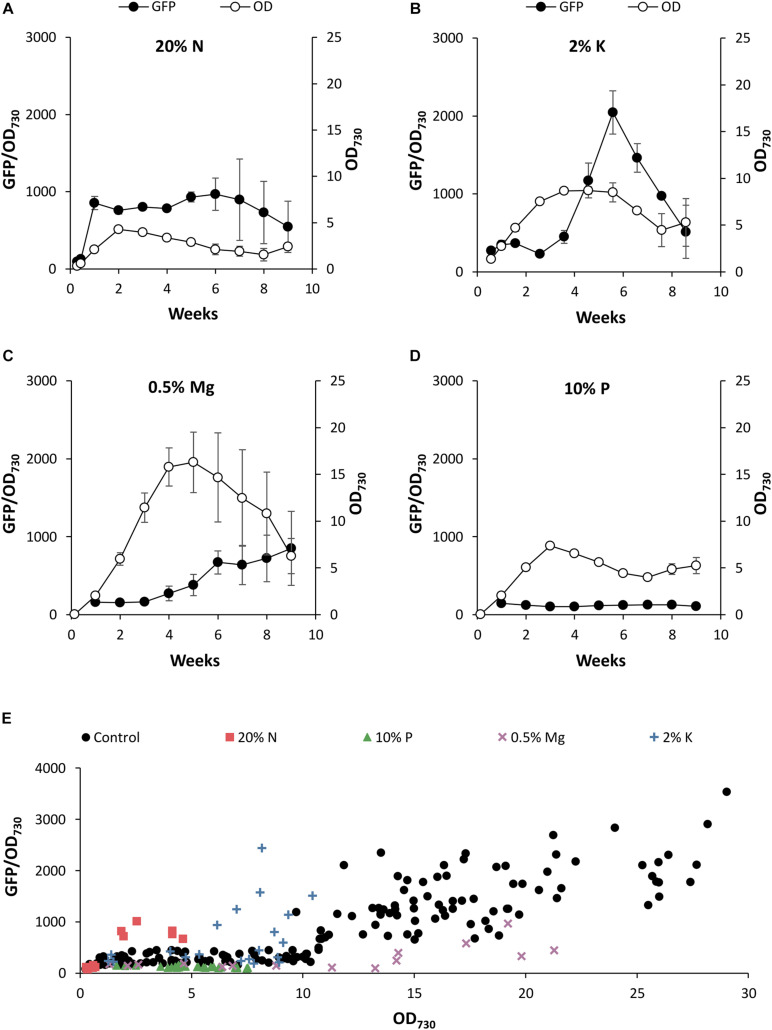
The effect of nutrient deficiency on PndbA600 activity. PndbA600 activity (black circles, GFP fluorescence normalised to OD_730_, left axis) in *Synechococcus* PndbA600:GFP cultures grown under low nutrient conditions (Supplementary Table S2). **(A)** 20% N, **(B)** 2% K, **(C)** 0.5% Mg, and **(D)** 10% P in A + background. Culture density (OD_730_, right axis) is shown with white circles. Data are means ± S.E.M. of three independent cultures. **(E)** PndbA600 activity (GFP fluorescence normalised to OD_730_) against culture density (OD_730_) in *Synechococcus* PndbA600:GFP grown in control (A+, black circles, *n* = 20) and low nutrient conditions (A + background; 20% N, red squares, *n* = 3; 10% P, green triangles, *n* = 3; 0.5% Mg, purple crosses, *n* = 3; 2% K, blue plusses, *n* = 3). Data represent measurements taken as culture density increased. Measurements taken after maximum culture density was achieved are not shown. For promoter activity under control conditions see Figure 4.

We have shown that the spent medium of stationary phase cultures is able to modulate PndbA600 activity by both inducing promoter activation and inhibiting promoter deactivation. A possible explanation for these responses may be the low nutrient levels in the spent stationary phase medium. The native expression profile in *Synechocystis* suggested that PndbA600 activation in the late growth phase occurs independent of nutrient depletion. However, this might not be the case in *Synechococcus*. We, therefore, investigated the nutrient dependence of PndbA600 activity in *Synechococcus* PndbA600:GFP using A + with low levels of individual nutrients. Counter ions co-supplied with nutrients were replaced up to the control concentration (Supplementary Table S2). Unlike the optimised BG11 medium for *Synechocystis*, we found that nutrient ratios in the standard A + medium used for *Synechococcus* are not optimally adjusted, and different nutrients become limiting at different concentrations (Figure 6). Timing of PndbA600 activation and, thus, correlations between the promoter activity, culture density, and the growth phase differed across nutrient conditions.

When comparing the promoter activity relative to the growth phase transition, promoter activation occurred during the transition to the stationary phase at > 75% of maximum culture density under control conditions (Figure 4A). Lowering N in the growth medium accelerated PndbA600 activation, whereby promoter activation occurred during the active growth phase at < 50% of maximum culture density (Figure 6A). By contrast, PndbA600 activation was delayed in media with low K or low Mg with promoter activation first occurring > 1 week after reaching maximum culture density (Figures 6B,C) and was completely abolished in low P (Figure 6D). In summary, PndbA600 activation, relative to the growth phase transition, is accelerated by lowering N and delayed by lowering other essential nutrients (and thus increasing the relative N level), suggesting that PndbA600 may be activated by the depletion of N relative to other nutrients in the media.

Plotting promoter activity directly against culture OD demonstrated promoter activation at high density (OD > 10.75) under control conditions (Figure 4B). Figure 6E shows that PndbA600 activation occurred at lower culture densities in media with 20% N (OD > 1.84) and 2% K (OD > 6.15). By contrast, PndbA activation required a much higher culture density (OD > 17) in media with 0.5% Mg. No PndbA activation was seen in 10% P at any OD. The varying culture OD requirements for PndbA activation under different low nutrient conditions reflect a combination of the maximum culture OD achieved and the timing of PndbA activation relative to the transition to stationary phase.

**FIGURE 7:**
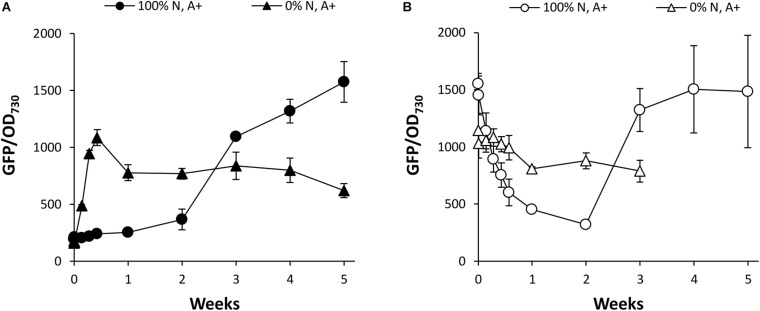
The effect of N supply on PndbA600 activity. PndbA600 activity (GFP fluorescence normalised to OD_730_) in *Synechococcus* PndbA600:GFP grown in media containing 0 or 100% N in A + background. Cultures shown in **(A)** were started from young, low density cultures with low promoter activity (OD < 5, GFP < 300, black symbols), and cultures shown in **(B)** were started from mature, high density cultures with high promoter activity (OD > 12, GFP > 850, white symbols). All cultures were resuspended to OD 1 in the indicated media at time point 0. Data are means ± S.E.M. of three independent cultures. Accompanying growth curves are presented in Supplementary Figure S6.

To investigate the effects of N supply in more detail, *Synechococcus* PndbA600:GFP cultures were grown in control conditions and resuspended at OD 1 in growth media containing either 0 or 100% N in a background of the control A + medium. Again, young *Synechococcus* PndbA600:GFP cultures grown to low density were used to investigate PndbA600 activation, and mature cultures grown to high density were used to investigate PndbA600 deactivation. Figure 7A confirms that PndbA600 activation requires the lack of N: early promoter activation was observed in N-deficient media (see also Supplementary Figure S6A). Similarly, PndbA600 deactivation requires the presence of N: mature, high density cultures resuspended at low density in N-deficient media maintained active levels of GFP fluorescence, albeit at a slightly lower level (Figure 7B and Supplementary Figure S6B). Combined, these results confirm that PndbA600 specifically responds to N levels, with promoter activation upon N depletion and promoter deactivation upon N replenishment.

### Early Kinetics of PndbA600 Activation

**FIGURE 8:**
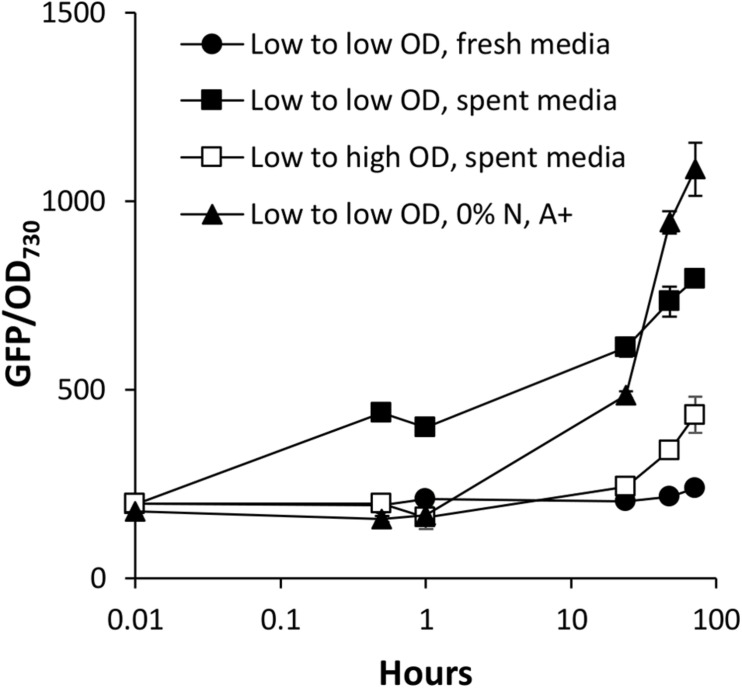
Early kinetics of PndbA600 activation. Early responses of PndbA600 in *Synechococcus* PndbA600:GFP to different treatments at time point 0: low OD < 5 to low OD 1 in fresh control medium (black circles), low OD < 5 to low OD 1 in spent medium (black squares), low OD < 5 to high OD 12 in spent medium (white squares), or low OD < 5 to low OD 1 in N-deficient medium (0% N in A + background, black triangles). Data are means ± S.E.M. of three independent cultures.

We have identified two types of media that accelerate the activation of PndbA600: the spent medium of stationary phase cultures and N-deficient medium. The spent medium is a complex solution comprised of a combination of different levels of multiple nutrient deficiency, as well as extracellular metabolites that have been secreted by the cells throughout culture growth. To check whether N depletion is the cause of PndbA600 activation in the spent medium, we compared PndbA600 activation in response to the spent medium with N-deficient medium (Figure 8). In the spent medium, PndbA600 activation is > 10-fold faster (within 30 min in low to low OD, spent media) compared to N-deficient medium (with 24 h in low to low OD, 0% N, A+). This suggests that nutrient deficiency, specifically N depletion, is not sufficient to explain the rapid activation in the spent medium and that other factor(s) contribute to PndbA600 activation.

### The Effect of Electron Transport Inhibition on PndbA600 Activity

**FIGURE 9:**
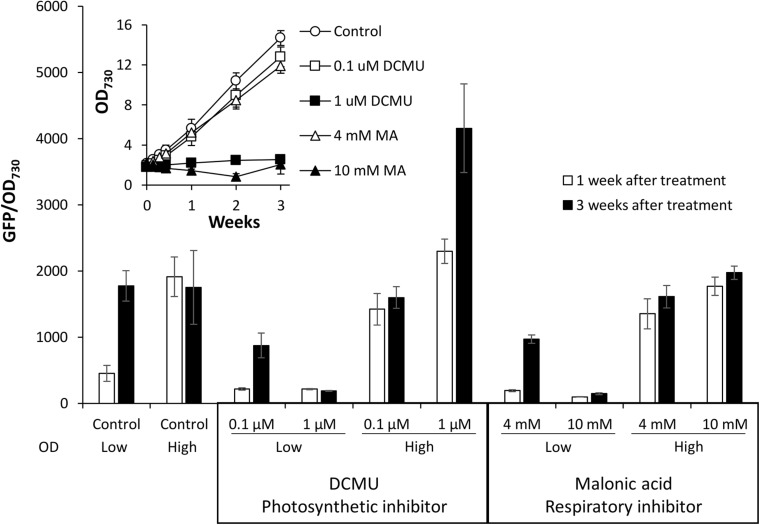
The effect of electron transport inhibition on PndbA600 activity. PndbA600 activity (GFP fluorescence normalised to OD_730_) in *Synechococcus* PndbA600:GFP 1 week (white bars) and 3 weeks (black bars) after treatment with DCMU or malonic acid at the given concentration. OD indicates low (OD < 5) or high (OD < 12) culture density at the start of treatment. Control are untreated cultures. Data are means ± S.E.M. of at least three independent cultures. Inset shows culture growth starting from low OD in the different conditions.

Type II NADH dehydrogenases play a central role in the respiratory metabolism of bacteria; however, this is not the case in cyanobacteria where NdbA function remains unclear (Howitt et al., 1999; Lea-Smith et al., 2016; Huokko et al., 2019). A role in redox sensing has been proposed (Howitt et al., 1999), so we tested the effect of changing cellular redox status on promoter activity by disturbing electron transport. For this experiment, electron transport inhibitors specific to photosynthesis [DCMU, which blocks the plastiquinone binding site of photosystem II (Duysens and Sweers, 1963)] or respiration [malonic acid (MA), which competitively inhibits succinate dehydrogenase complex II (Pardee and Potter, 1949)] were applied to low or high density cultures of *Synechococcus* PndbA600:GFP grown under control conditions. Inhibitors were applied at two concentrations: a lower concentration, which allowed for growth of low density cultures (0.1 μM DCMU and 4 mM MA), and a high concentration, which inhibited the growth of low density cultures (1 μM DCMU and 10 mM MA; Figure 9). When the inhibitors were applied at low concentration in young, low density cultures, PndbA600 activation in the late growth phase still occurred, albeit at a lower level than without inhibitor. The slight decrease in activity may reflect the slower growth and lower density of the inhibitor-treated cultures. By contrast, high inhibitor concentrations resulted in a lack of culture growth and of PndbA600 activation. In mature, high density cultures, low concentrations of inhibitors did not alter PndbA600 activity. At high concentrations, however, DCMU increased PndbA600 activity 1.79-fold in high density cultures whereas MA did not. These results show that the inhibition of photosynthesis, but not respiration, enhances PndbA600 activity in high density cultures.

## Discussion

Two-stage cultivation strategies are an attractive solution to growth/productivity trade-offs in cyanobacteria; however, the costly addition of extra steps between growth (stage I) and production (stage II) is often required to initiate stage II (Lee et al., 2012, 2016; Monshupanee et al., 2016; Kushwaha et al., 2018; Aziz et al., 2020). Less effort has been made towards utilising inherent features of cyanobacterial cultures to distinguish between stages and thus auto-induce transgene expression in stage II, cutting costs and improving economic feasibility. Auto-inducible production systems have been engineered using nutrient-deficiency responsive regulators (Liu et al., 2011; Asada et al., 2019); however, their applications may be limited by the regulatory nutrient and the timing of deficiency. Here, we instead opted to develop regulatory systems based on growth phase transitions in batch cultures and endogenous regulation by stationary phase promoters. A few studies led to the identification of cyanobacterial growth phase-responsive genes and promoters (Foster et al., 2007; Berla and Pakrasi, 2012; Ruffing et al., 2016); however, the knowledge is still very limited for the stationary phase and its regulation in cyanobacteria. In this study, we identified a small subset of genes that specifically respond to growth phase transition in *Synechocystis* sp. PCC 6803. Furthermore, we report the first detailed description of the complex environmental responses of a growth phase-responsive promoter of cyanobacteria.

### General Responses to Changes in Growth Phase

Common responses of nutrient limitation studies form the main foundation of knowledge surrounding stationary phase in cyanobacteria and generally involve increased catabolism and decreased anabolism (Schwarz and Forchhammer, 2005). Direct comparisons of cyanobacterial growth phases have focussed on transcriptional responses in at most two conditions (Foster et al., 2007; Ludwig and Bryant, 2011; Berla and Pakrasi, 2012; Kopf et al., 2014). These studies have led to the identification of differentially expressed genes; however, it is difficult to differentiate between genes involved in general responses to changes in the growth phase and specific responses to the conditions in which the cultures were grown. Furthermore, it is often difficult to confirm that these datasets do in fact reflect stationary phase gene expression as low stationary phase ODs are commonly reported without any accompanying growth curves. Here, we present a comprehensive RNA sequencing dataset that enables the robust separation of growth phase-specific responses from condition-specific responses in *Synechocystis* sp. PCC 6803 (Supplementary Table S3). Our dataset agrees with general, growth phase-related downregulation of genes involved in photosynthesis, energy metabolism, and translation reported by previous nutrient limitation studies (Hirani et al., 2001; Richaud et al., 2001). Of the genes upregulated in the late growth phase/early stationary phase, 67% are annotated as hypothetical proteins reflecting a large gap in knowledge that persists on growth phase-specific responses in the extensively studied model cyanobacterium *Synechocystis* sp. PCC 6803. Besides their purpose for this study, the datasets provided in Supplementary Table S3 represent a new resource for understanding transcriptional responses of *Synechocystis* sp. PCC 6803 to individual nutrient deficiencies (including new transcriptomes in low Mg and low K).

### Orthogonal Promoter Behaviour in Cyanobacteria

Orthogonal molecular parts are preferred for metabolic engineering in order to avoid the interference of engineered systems by host machinery and genetic instability arising from endogenous DNA sequences (Camsund and Lindblad, 2014). Well-established prokaryotic tools such as IPTG- and tetracycline-inducible systems from *E. coli* perform poorly in cyanobacteria (Huang et al., 2010). Promoters derived from other species of cyanobacteria appear to have greater success despite regulatory differences between cyanobacterial species (Huang et al., 2010; Wang et al., 2012; Gordon and Pfleger, 2018). For example, the phycocyanin promoters (Pcpc) of *Synechocystis* sp. PCC 6803 and PCC 6714 have been used to drive heterologous expression in *Synechococcus* sp. PCC 7002 and PCC 7942 (Markley et al., 2015; Gao et al., 2016). Furthermore, a previous report showing higher activity of the heterologous phycocyanin promoter in the early growth phase of *Synechococcus* sp. PCC 7002 agrees with the expression profiles of the native phycocyanin operon in our *Synechocystis* sp. PCC 6803 RNAseq dataset (Madsen et al., 2018). Nevertheless, our detailed analysis of heterologous PndbA600 activity in *Synechococcus* sp. PCC 7002 revealed clear differences to the endogenous *ndbA* expression profiles in *Synechocystis* sp. PCC 6803. While expression patterns were similar under control conditions (Figures 3B,C, 4A,B), closer examination under nutrient limitation revealed differences in correlations between the gene expression, culture density, and the growth phase in the two species (Figure 6). The promoter activation during the late growth phase observed under control conditions in this study is not an artefact of the assay, as evidenced by previous characterisation of an early growth phase-specific promoter using the same approach (Madsen et al., 2018). While this suggests that the condition-dependent activation profiles of PndbA600 should reflect promoter response to growth phase status, further studies using a truncated version of PndbA600, which has lost the ability to respond to changes in the growth phase, will strengthen this finding. This study, therefore, highlights the importance of thorough characterisation of molecular components to enable rational design and accurate prediction of the behaviour of more complex assemblies in non-standard conditions.

### Environmental Regulation of PndbA600

**FIGURE 10:**
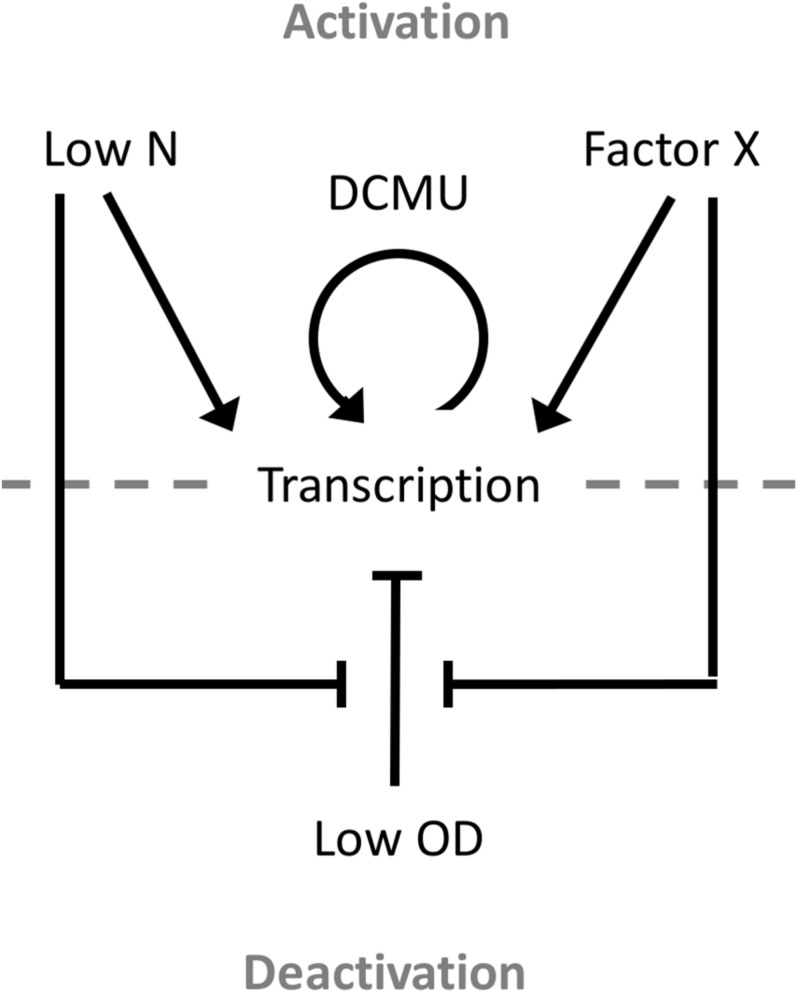
Model of the environmental regulation of PndbA600. Promoter activation and, thus, transcription occur in response to either low N or Factor X of the spent stationary phase medium. Once activated, promoter activity can be further increased using the photosynthesis-specific inhibitor DCMU. Promoter deactivation requires low culture density, sufficient N supply, and the absence of Factor X.

This study also presents the first detailed analysis of a growth phase-responsive promoter of cyanobacteria. Responses to changing environmental stimuli, including culture density, growth media, nutrient availability, and cellular redox status, showed differing requirements for PndbA600 activation and deactivation (Figure 10). PndbA600 activation could be induced either by lowering N supply or presenting Factor X from the spent medium of stationary phase cultures. Interestingly, culture density *per se* had no effect on PndbA600 activation, but low culture density was required for PndbA600 deactivation in addition to sufficient N supply and the absence of Factor X. Furthermore, PndbA600 may respond in a dose-dependent manner to Factor X. Increasing the relative amount of Factor X per cell may have a proportionate effect on PndbA600 activity. For example, PndbA600 activation in spent media was > 100-fold faster in young cultures resuspended at low density compared to high density (Figures 5B, 8). PndbA600 activity in spent media was also higher in mature cultures resuspended at low density compared to high density (Figure 5D). Similarly, mature cultures transferred from spent to N-deficient media showed a visible reduction in GFP fluorescence, which could be due to the removal of Factor X (Figure 7B). Additional analyses are required to identify Factor X, which could be either a downstream response to N limitation or an unrelated extracellular metabolite e.g., signalling molecule secreted by stationary phase cells. Compositional analyses of spent media fractions paired with gene expression and promoter analyses could yield further insights into stationary phase and its regulation in cyanobacteria.

While heterologous promoter activity may not accurately reflect endogenous transcriptional regulation, this study could provide initial insights into growth phase-specific regulation and NdbA function in cyanobacteria. The *ndbA* gene encodes a type II NADH dehydrogenase of unknown function. The ability of *ndbA* knockout strains of *Synechocystis* sp. PCC 6803 to grow under otherwise lethal high-light conditions in a PSI-less background led to the suggestion of a regulatory role for NdbA and monitoring of, e.g., cellular redox status (Howitt et al., 1999). In support of these functions, native NdbA localises to the thylakoid membrane (Huokko et al., 2019), and the PndbA600 promoter responds to disturbances in electron transport in the heterologous expression host (Figure 9). Specifically, heterologous PndbA600 responds to photosynthetic, but not respiratory, inhibition, whereby the key difference is the reduction of NADP + in photosynthesis and oxidation of NADH in respiration. These findings, paired with the presence of NADH binding motifs in the *ndbA* coding sequence (Howitt et al., 1999), suggest that NAD(P) + /NAD(P)H balance may be an important factor regulating PndbA600/NdbA activity levels.

### Industrial Applications

This study describes first steps towards developing regulatory systems to drive stage II of a two-stage cultivation system in cyanobacteria. Libraries of stage II promoters can now be constructed based on PndbA600 or other late growth phase-responsive promoters and, subsequently, used to optimise heterologous metabolic pathways for industrial production or fine-tune endogenous metabolic pathways supplying precursors necessary for the engineered process. Similarly, libraries of stage I promoters can be constructed to optimise growth, potentially improving growth rates and thus decreasing time scales until the initiation of stage II. Furthermore, many additional analyses can be performed on the RNAseq dataset to identify genes/promoters with any combination of growth phase- and/or nutrient-specific activity for diverse applications in industry, e.g., biosensors.

Stationary phase may not be suitable for all industrial applications or commercial products, and therefore, a careful selection of products and processes is important. While general decreases in anabolism occurs during the stationary phase, select processes continue at appreciable levels even after prolonged starvation (Schwarz and Forchhammer, 2005). Notable examples are secondary metabolites important for human health, particularly as anti-infective drugs such as antibiotics (Ruiz et al., 2010). Here, we used a new approach for the robust separation of growth phase- vs. condition-specific processes. This approach can also complement bioprospecting for new secondary metabolites in cyanobacteria and other microorganisms by comparing transcriptomic and metabolomic data across a variety of conditions to identify genes and unravel biosynthetic pathways underpinning the production of interesting metabolites. The large proportion (67%) of late growth phase-specific genes encoding hypothetical proteins identified in *Synechocystis* sp. PCC 6803 highlights the strength of this approach, as well as the great potential for the identification of new cyanobacterial products and pathways.

As a chassis, *Synechococcus* sp. PCC 7002 has numerous advantages for industrial production, including relatively fast growth rates and high tolerance to various parameters such as light, temperature, and salinity (Nomura et al., 2006). Another desirable feature we have observed in this strain is sedimentation in the stationary phase, which allows for easy biomass harvest at the end of stage II without the need for energy-demanding techniques such as centrifugation (data not shown). Perhaps, the most sustainable application of PndbA600-driven two-stage cultivation strategies involves seeding the engineered *Synechococcus* cultures in the fresh growth medium, biomass accumulation during stage I until nutrient depletion results in the auto-induction of stage II, and finally application-specific downstream processing of the biomass and supernatant. Recycling stage II cells to seed new cultures is not ideal as we observed a decrease in the amount of biomass attained, no increase in promoter activity, and no decrease in time to stage II (high to low OD in fresh media, Supplementary Figure S5C). By contrast, maximum biomass can be further increased by concentrating low density cultures in fresh nutrients to generate higher culture densities compared to control conditions (low to high OD in fresh media, Supplementary Figure S5A). Alternatively, if the expense of time is greater than the benefit of high biomass, stage II can be induced at lower culture densities by using nutrient limitation to significantly reduce timescales. Early induction could prove particularly profitable if nutrient-specific responses increase the productivity of stage II cells. Finally, stage II productivity can be further improved by increasing the activity of the auto-inducible promoter, either by engineering PndbA600 or adding supplements such as DCMU.

This study provides the first insights into the regulation of stationary phase in cyanobacteria. Additional studies to identify DNA motifs present within growth phase-responsive promoters, transcription factors that bind to these motifs, and other regulatory molecules will provide further important insights to this still elusive phase of cyanobacteria. Unravelling these mysteries and expanding the foundation of knowledge surrounding these organisms will be of great value to both academia and industry.

## Data Availability Statement

The datasets presented in this study can be found in online repositories. The names of the repository/repositories and accession number(s) can be found below: https://www.ebi.ac.uk/ena, PRJEB40560.

## Author Contributions

MAM and AA designed the study and wrote the manuscript. MAM performed the experiments. MAM, GH, PH, and AA analysed the data. All authors contributed to the article and approved the submitted version.

## Conflict of Interest

The authors declare that the research was conducted in the absence of any commercial or financial relationships that could be construed as a potential conflict of interest.
