# Supplementary material for: Environmental Regulation of PndbA600, an Auto-Inducible Promoter for Two-Stage Industrial Biotechnology in Cyanobacteria
Source: Front Bioeng Biotechnol. 2021 Jan 19;8:619055. doi: 10.3389/fbioe.2020.619055 (PMC7853294; doi:10.3389/fbioe.2020.619055)
Supplement: Supplementary file 1 [file Data_Sheet_1.docx]

Supplementary Material

# Supplementary Data

All data represented in the figures can be found in Additional file **Data sheet 2.ZIP**.

# Supplementary Figures and Tables

## Supplementary Figures


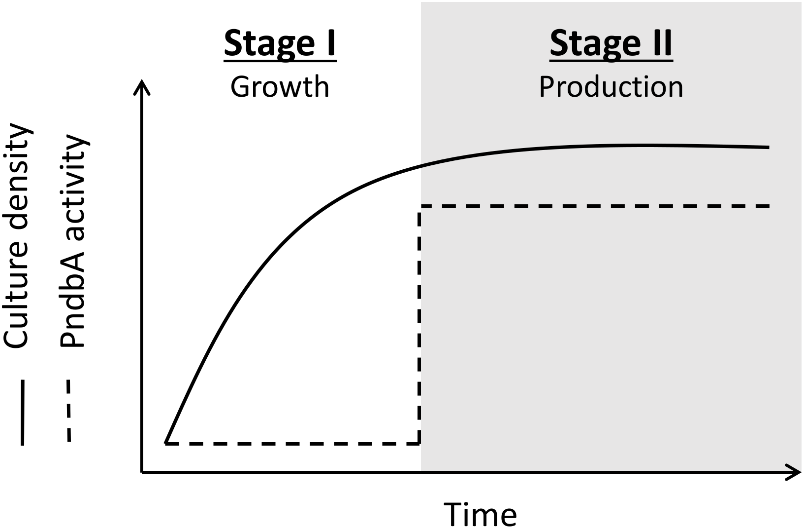


**Figure S1**. **Schematic of a two-stage batch cultivation strategy.** Stage I is shown in white followed by stage II in grey. The solid line represents batch culture density. The dashed line represents promoter activity.

**Figure S2**. **Map of integrative transformation vector pAQ1BB:PndbA600:RBS3:GFP.** Vector used for transformation of *Synechococcus* sp. PCC 7002 to generate the transgenic *Synechococcus* PndbA600:GFP strain. Region between Flanks A and B, which stably integrates into the native pAQ1 *Synechococcus* plasmid, carries the 600 bp upstream sequence of *ndbA* (PndbA600), synthetic ribosome binding site RBS3, GFP BioBrick BBa_E0040, double T1/TE terminator BioBrick BBa_B0015, and spectinomycin selective marker gene *aadA*. The f1 origin of replication and ampicillin resistance gene AmpR are for propagation and selection in *E. coli*, respectively. Primer binding sites pAQ1BB-seq-F and pAQ1BB-seq-R are for sequence confirmation. Restriction sites EcoRI, XbaI, SpeI and PstI are for BioBrick assembly.

| GAATTCGCGGCCGCTTCTAGAG**TTAATGGATCGTTACCATTCCCACACTG** | 50 |
| --- | --- |
| **AACATTGCCACAGTTGCCGCAATGCCCTTGCTAACATTGAAAAAATTCAA** | 100 |
| **ACCGGGAGCCTCGCGATCGCCATTATCTGCTTGGTTTGCTTGCCAGTGGC** | 150 |
| **TAGTTTAGGCTTAGCTCCTAATTTCGCTCTGGGATTGGGCTTGACAGTGT** | 200 |
| **TGACTGGCTTAGGTTTTGCACTCTGGGCTGGTTTGGGCAAATTTAAACAA** | 250 |
| **TCTTTTTACCAAGGCAACCCCATTCCCCCAAGAAATATTCTTGATAAAGG** | 300 |
| **CTAACTGCTTTTTTCTGTGATTAAAAACCTTTGCAATGGAGTATTGAGCA** | 350 |
| **TAATTTTATTGCCGAAAGTAAGACCATTAAACTGAATCTAAGAGCCTGTT** | 400 |
| **TAAAAGCCCCCTTGACCCCCAAATTTGGGGGGACACTGACTGAAAGTCCC** | 450 |
| **CCAGTATTGCCGGAGCCTTAGTGAGTTAATTTAGAGGATGGAATAAAACT** | 500 |
| **TTCCAAACACGTTCCAACGTCAGGGTTTGGCCAAGAATAATCGATATCTT** | 550 |
| **TGTAACAACCATTACCGGTAATCCCCAAGGAAATGATTACCATTAACAAA** | 600 |
| **TCGTAATATTTTCGCCGTTGCT**TACTAGAG**AGAATTACATATCGCACAAA** | 650 |
| **GGTATATAAAA**ATGCGTAAAGGAGAAGAACTTTTCACTGGAGTTGTCCCA | 700 |
| ATTCTTGTTGAATTAGATGGTGATGTTAATGGGCACAAATTTTCTGTCAG | 750 |
| TGGAGAGGGTGAAGGTGATGCAACATACGGAAAACTTACCCTTAAATTTA | 800 |
| TTTGCACTACTGGAAAACTACCTGTTCCATGGCCAACACTTGTCACTACT | 850 |
| TTCGGTTATGGTGTTCAATGCTTTGCGAGATACCCAGATCATATGAAACA | 900 |
| GCATGACTTTTTCAAGAGTGCCATGCCCGAAGGTTATGTACAGGAAAGAA | 950 |
| CTATATTTTTCAAAGATGACGGGAACTACAAGACACGTGCTGAAGTCAAG | 1000 |
| TTTGAAGGTGATACCCTTGTTAATAGAATCGAGTTAAAAGGTATTGATTT | 1050 |
| TAAAGAAGATGGAAACATTCTTGGACACAAATTGGAATACAACTATAACT | 1100 |
| CACACAATGTATACATCATGGCAGACAAACAAAAGAATGGAATCAAAGTT | 1150 |
| AACTTCAAAATTAGACACAACATTGAAGATGGAAGCGTTCAACTAGCAGA | 1200 |
| CCATTATCAACAAAATACTCCAATTGGCGATGGCCCTGTCCTTTTACCAG | 1250 |
| ACAACCATTACCTGTCCACACAATCTGCCCTTTCGAAAGATCCCAACGAA | 1300 |
| AAGAGAGACCACATGGTCCTTCTTGAGTTTGTAACAGCTGCTGGGATTAC | 1350 |
| ACATGGCATGGATGAACTATACAAATAATAATACTAGAG*CCAGGCATCAA* | 1400 |
| *ATAAAACGAAAGGCTCAGTCGAAAGACTGGGCCTTTCGTTTTATCTGTTG* | 1450 |
| *TTTGTCGGTGAACGCTCTCTACTAGAGTCACACTGGCTCACCTTCGGGTG* | 1500 |
| *GGCCTTTCTGCGTTTATA*TACTAGTAGCGGCCGCTGCAG | 1539 |

**Figure S3**. **DNA sequence of promoter-reporter expression construct PndbA600:RBS3:GFP.** 5’ to 3’ DNA sequence comprised of BioBrick prefix (nucleotides 1-22), 600 bp upstream sequence of *ndbA* (PndbA600, bold underlined, nucleotides 23-622), synthetic ribosome binding site (RBS3, bold, nucleotides 631-661), GFP reporter gene (BioBrick BBa_E0040, underlined, nucleotides 662-1381), double T1/TE terminator (BioBrick BBa_B0015, italics, nucleotides 1390-1518), and BioBrick suffix (nucleotides 1519-1539). Nucleotide position is indicated to the right of the sequence.


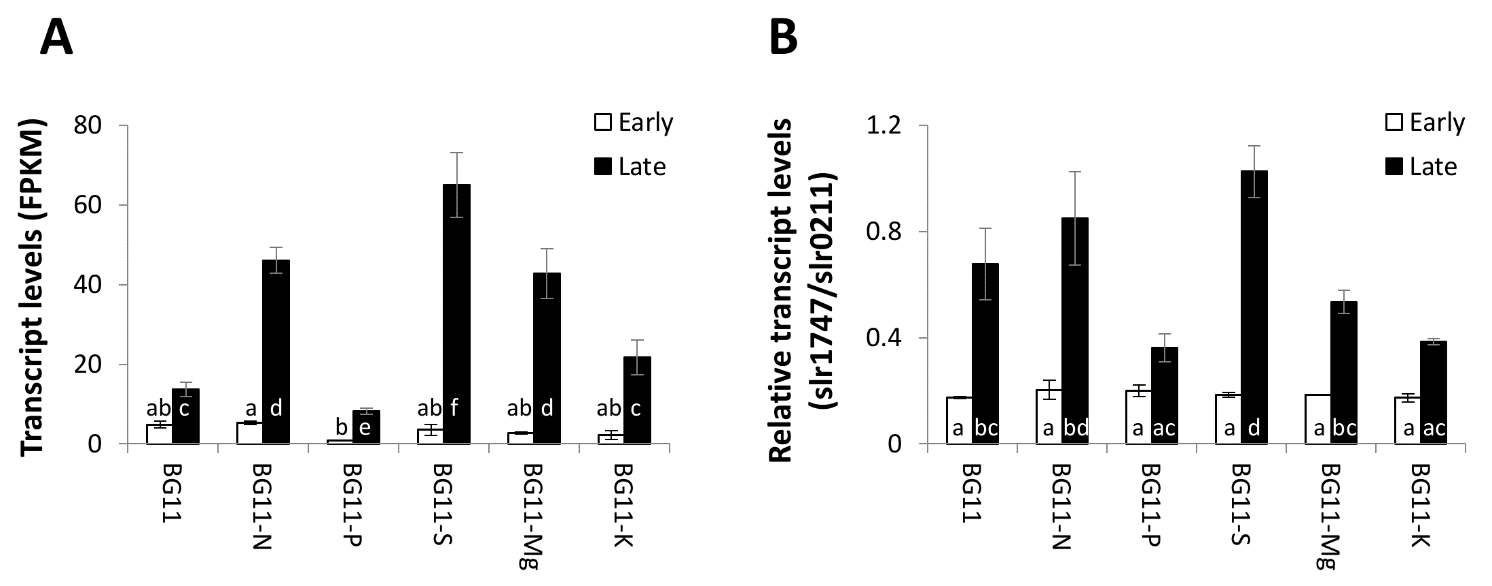


**Figure S4.** **Native *slr1747* transcript levels.** Transcript levels of *slr1747* determined by **(A)** RNA sequencing (normalised to gene length and read counts as FPKM) and **(B)** qPCR (normalised to *slr0211*) in early (white bars) and late (black bars) growth phases of *Synechocystis* cultivated under control (BG11) and low nutrient conditions (12.5% N, P, S, Mg or K in BG11 background). Data are means ± S.E.M. of three independent cultures. Different letters indicate significant difference across all conditions (p<0.05; two-way ANOVA using Tukey (HSD) post-hoc analysis).


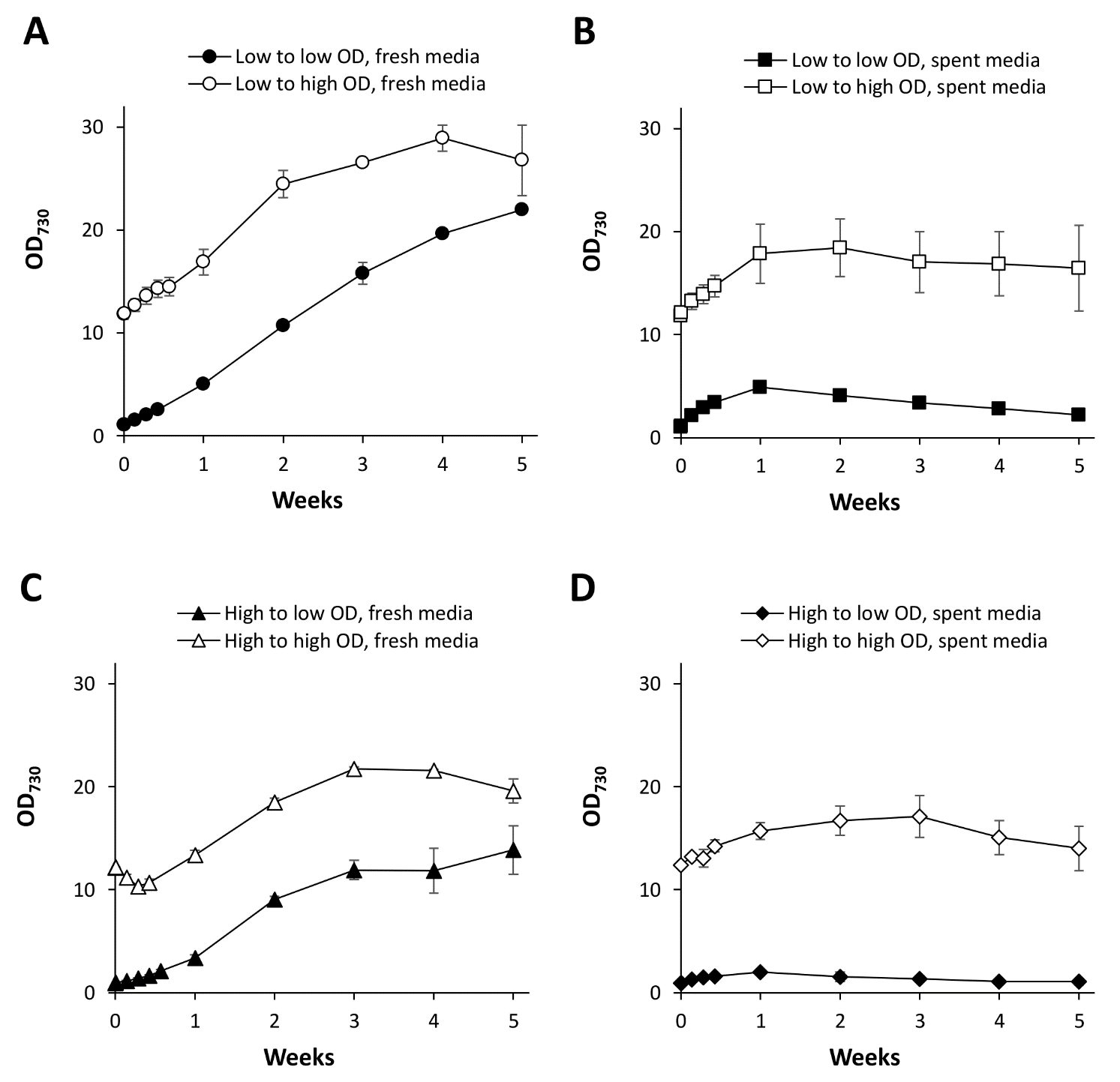


**Figure S5.** **Growth curves of cell density experiments.** Culture density (OD_730_) of *Synechococcus* PndbA600:GFP cultures pre-grown to certain density and resuspended to another density at time point 0. Cultures shown in **(A,B)** were started from young, low density cultures (OD <5). Cultures shown in **(C,D)** were started from mature, high density cultures (OD >12). Cultures were resuspended to low (black symbols) or high (white symbols) density in fresh control media **(A,C)** or spent media of stationary phase cultures **(B,D)**. Data are means ± S.E.M. of three independent cultures. Accompanying promoter activity curves are presented in **Figure 5**.


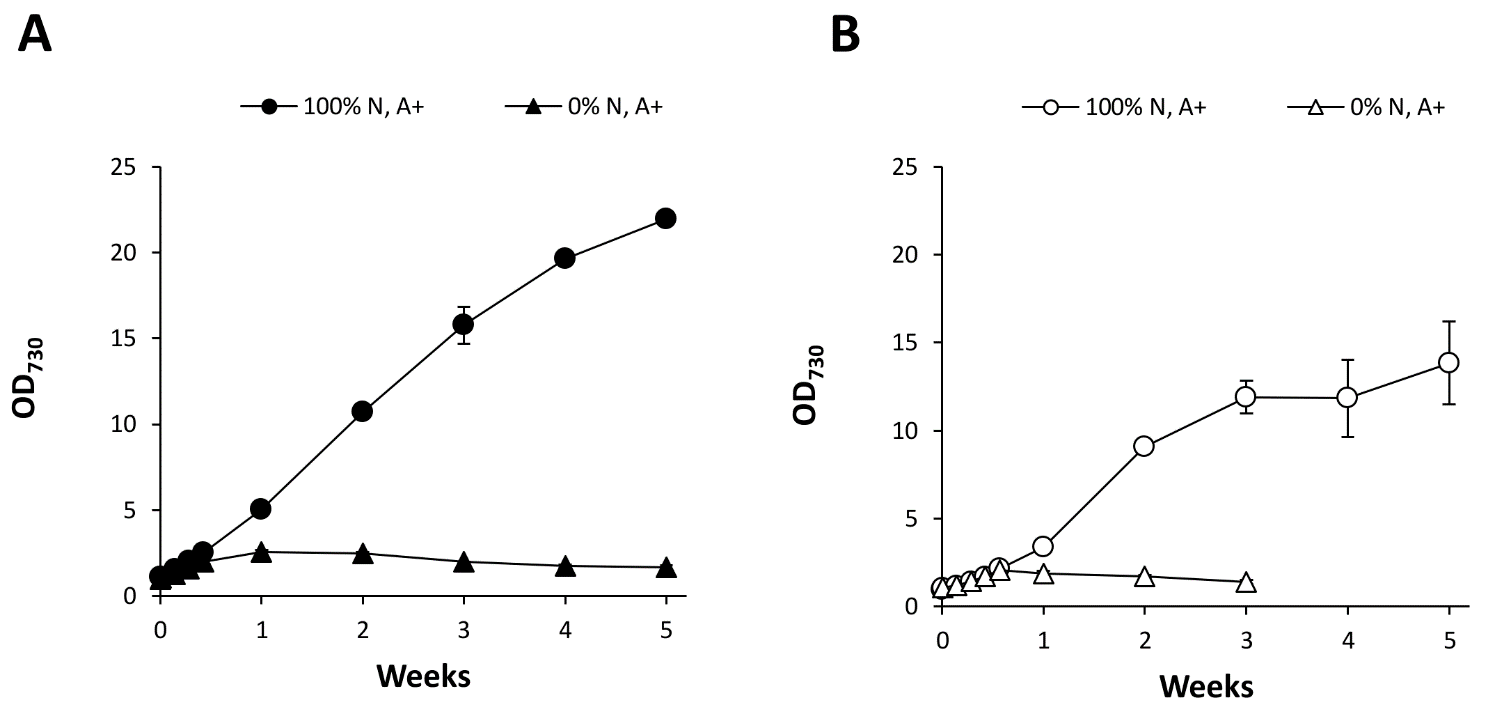


**Figure S6.** **Growth curves of N supply experiments.** Culture density (OD_730_) of *Synechococcus* PndbA600:GFP cultures grown in media containing 0% or 100% N in A+ background. Cultures shown in **(A)** were started from young, low density cultures (OD <5, black symbols), cultures shown in **(B)** were started from mature, high density cultures (OD >12, white symbols). All cultures were resuspended to OD 1 in the indicated media at time point 0. Data are means ± S.E.M. of three independent cultures. Accompanying promoter activity curves are presented in **Figure 7**.

## Supplementary Tables

**Table S1. Low nutrient conditions in BG11 background for *Synechocystis* sp. PCC 6803.**

| **Condition** | **Nutrient compound** | **Control concentration** | **Low concentration** | **Replacement counter ion compound** |
| --- | --- | --- | --- | --- |
| BG11-N | NaNO_3_ | 17.6 mM | 2.2 mM | None |
| BG11-P | K_2_HPO_4_ | 230 µM | 28.7 µM | KCl |
| BG11-S | MgSO_4_ | 304 µM | 38 µM | MgCl |
| BG11-Mg | MgSO_4_ | 304 µM | 38 µM | Na_2_SO_4_ |
| BG11-K | K_2_HPO_4_ | 230 µM | 28.7 µM | NaH_2_PO_4_ |

**Table S2. Low nutrient conditions in A+ background for *Synechococcus* sp. PCC 7002.**

| **Condition** | **Nutrient compound(s)** | **Control concentration** | **Low concentration** | **Replacement counter ion compound** |
| --- | --- | --- | --- | --- |
| 20% N | NaNO_3_ | 11.8 mM | 2.36 mM | None |
| 10% P | KH_2_PO_4_ | 367 µM | 36.7 µM | KCl |
| 0.5% Mg | MgSO_4_ | 20.3 mM | 0.1 mM | Na_2_SO_4_ |
| 2% K | KCl, KH_2_PO_4_ | 367 µM | 7.34 µM | NaH_2_PO_4_ |

**Table S3. Normalised transcript levels with statistical parameters for the RNA sequencing** **dataset comparing** **growth phases and nutrient conditions in wildtype *Synechocystis* sp. PCC 6803.** Growth media were control (BG11) and low nutrient (12.5% N, P, S, Mg or K in BG11 background). The table displays the following for each gene: gene annotation according to Cyanobase, genomic context, normalised transcript levels for each sample determined by RNA sequencing (FPKM), and all pairwise comparisons determined using Cuffdiff software. Conditions are control conditions (BG11), low nitrate (N), low phosphate (P), low sulphate (S), low magnesium (Mg) and low potassium (K). Time points are day 4 (D4) representing early growth phase and day 8 (D8) or 16 (D16) representing late growth phase in low nutrient and control conditions respectively. The raw RNA sequencing data are available from the European Nucleotide Archive, accession number PRJEB40560. [Additional file **Table 3.XLSX**]
